# Novel Gluten-Free Cinnamon Rolls by Substituting Wheat Flour with Resistant Starch, Lupine and Flaxseed Flour

**DOI:** 10.3390/foods11071022

**Published:** 2022-03-31

**Authors:** Sofyan Maghaydah, Asma Alkahlout, Mahmoud Abughoush, Nazieh I. Al Khalaileh, Amin N. Olaimat, Murad A. Al-Holy, Radwan Ajo, Imranul Choudhury, Waed Hayajneh

**Affiliations:** 1 Department of Nutrition and Food Technology, Faculty of Agriculture, Jordan University of Science and Technology, Irbid 22110, Jordan; maghaydah@just.edu.jo (S.M.); asmaalkahlout91@gmail.com (A.A.); waed41@gmail.com (W.H.); 2 Department of Clinical Nutrition and Dietetics, Faculty of Applied Medical Sciences, The Hashemite University, Zarqa 13133, Jordan; aminolaimat@hu.edu.jo (A.N.O.); murad@hu.edu.jo (M.A.A.-H.); 3 Science of Nutrition and Dietetics Program, College of Pharmacy, Al Ain University, Abu Dhabi P.O. Box 64141, United Arab Emirates; 4 Department of Nutrition and Food Science, Faculty of Agriculture, Mu’tah University, Karak 61710, Jordan; nazieh@mutah.edu.jo; 5 Nutrition and Food Processing Department, Al-Huson University College, Al-Balqa Applied University, Al-Huson 21510, Jordan; radwan_ajo@bau.edu.jo; 6 College of Pharmacy, Al Ain University, Abu Dhabi P.O. Box 64141, United Arab Emirates; imranul.haq@aau.ac.ae

**Keywords:** celiac disease, lupine flour, resistant starch, flaxseed flour, gluten-free cinnamon rolls

## Abstract

Celiac disease (CD) is an immunological mediated disorder that occurs to genetically susceptible individuals who suffer from gluten consumption. Therefore, the most effective treatment of CD is a life-long gluten-free diet. This study aimed to produce a nutritious gluten-free cinnamon roll, where resistant starch and lupine flour were used instead of wheat flour, in addition to 10% flaxseed flour and a fixed amount of hydrocolloid (1% xanthan gum). Eight different gluten-free cinnamon roll treatments (T1–T8) were produced with different ratios of resistant starch and lupine flour according to the following percentages (85:5, 80:10, 75:15, 70:20, 65:25, 60:30, 55:35 and 50:40, respectively). The proximate analysis, physical properties, color measurements and sensory evaluation of all cinnamon roll treatments and flours were determined. It was found that lupine and flaxseed flours in all different treatments had significantly (*p* ≤ 0.05) higher levels of ash, protein, lipid and crude fiber compared to wheat flour treatment (control treatment). However, carbohydrate levels were significantly (*p* ≤ 0.05) higher in control treatment compared with treatments 3–8. Gluten-free cinnamon rolls had significantly (*p* ≤ 0.05) higher levels of unsaturated fatty acids (oleic acid, linoleic acid and linolenic acid) than control. Further, there were significant differences in lightness (L*), redness (a*) and yellowness (b*) color values between the gluten-free and control treatments. The control cinnamon roll significantly (*p* ≤ 0.05) had the highest level of lightness and the lowest level of redness. The sensory evaluation obtained by consumer evaluation indicated that control cinnamon rolls significantly (*p* ≤ 0.05) received the highest score in overall impression, overall flavor, hardness and aftertaste. However, treatment 5 significantly (*p* ≤ 0.05) received the highest score in all the sensory scores in comparison with other gluten-free treatments. It is possible to develop a quality gluten-free cinnamon roll with respect to nutritional value manifested in higher levels of protein, fibers, unsaturated acids and prebiotics with acceptable sensory attributes.

## 1. Introduction

Celiac disease (CD) is a chronic systemic autoimmune disorder caused by intolerance of gluten proteins in susceptible individuals; it is characterized by various gastrointestinal symptoms such as diarrhea, steatorrhea, abdominal pain and severe malabsorption of macro and micronutrients [1]. Strict avoidance of gluten, found in wheat, barley, ray and oats, would result in intestinal healing and symptom relief. Gluten is a protein complex comprised of two main fractions—gliadin and glutenin—formed when wheat flour is sufficiently hydrated [2]. Gliadin and glutenin fractions are combined by covalent and non-covalent bonds to form gluten complex protein [3]. However, gliadin and glutenin in CD are responsible for the toxicity of the intestinal mucosa, but gliadin is the main toxic fraction and, therefore, the body’s immune system responds abnormally to gluten, resulting in damage of the small intestine and reduction in absorption of various minerals such as iron and calcium and vitamins such as A, D, E, K and folate [4,5]. Currently, a strict lifelong gluten-free diet (GFD) is the only treatment for CD and other gluten-related disorders [6,7]. However, it is demonstrated that wheat products are the major consumption products in diets worldwide [8], therefore, exclusion of wheat products is a critical challenge for CD patients. Cinnamon rolls consist of a sheet of yeast-leavened dough onto which a cinnamon and sugar mixture is sprinkled over a thin coat of butter. The wheat flour in this product could be replaced by other types of gluten-free flour with similar functional properties to that of gluten. A wide variety of alternative flours could be used such as lupine flour, resistant starch and flaxseed flour. Lupine flour could be used in bakery products due to its nutritional and functional properties which contain a large amount of protein (35–40%), fiber (up to 39%) and unsaturated fatty acid (85–90% of total fat) and a small amount of carbohydrate [9]. Moreover, resistant starch is a functional indigestible starch in the small intestine [10]. Resistant starch is considered as a prebiotic fermented by colonic bacteria to produce gases and short-chain fatty acids such as butyrate (the main fuel for colon cells) that reduce a number of colonic diseases [11]. Flaxseed flour is one of the most healthful gluten-free flours since it contains high levels of fiber (28%), protein (17.91%), fat (37.28%) [12] and carbohydrate (20.70%) [13]. Further, it contains a high amount of omega 3, 6 and 9 fatty acids (57%, 16% and 18% of unsaturated fatty acids, respectively) which could decrease the risk of heart disease, as well as a high content of lignin that would reduce the risk of breast cancer [14].Currently, there is no local production of gluten-free cinnamon rolls and it is necessary to develop such a product at an affordable price. Consequently, the main objective of this study was to develop a healthier gluten-free cinnamon roll higher in protein, fiber, unsaturated fatty acids and prebiotics with acceptable sensory attributes.

## 2. Materials and Methods

### 2.1. Flour

Zahra wheat flour (extraction rate 45%), sweet lupine flour (*Lupinus albus*) and flaxseed flour were obtained from a local market in Jordan. Resistant starch was obtained from Cargill Company, France. The wheat flour, resistant starch, lupine and flaxseed flours were obtained in various weight as 50 kg, 5 kg, 50 kg and 0.5 kg, respectively. Flour treatments were stored in tight polyethylene bags and kept at room temperature that ranged from 24–28 °C until analysis.

### 2.2. Other Cinnamon Roll Ingredients

Soy oil, butter, sugar, milk, egg, vanilla, sodium bicarbonate, yeast, walnuts, ground cinnamon, concentrated milk and xanthan gum were obtained from a local market in Jordan.

### 2.3. Cinnamon Roll Preparation

Cinnamon roll processing was carried out at the food processing factory at Jordan University of Science and Technology, according to Fernandez [15], with some modification. Eight different gluten-free cinnamon roll treatments (T1–T8) were produced with different ratios from resistant starch and lupine flour according to the following percentages: 85:5, 80:10, 75:15, 70:20, 65:25, 60:30, 55:35 and 50:40, respectively. In addition to 10% flaxseed flour and a fixed amount of hydrocolloid (1% xanthan gum). The ingredients used in control cinnamon roll production are shown in Table 1, Table 2 and Table 3.

#### 2.3.1. Mixing

Mixing of cinnamon roll dough ingredients was divided into two parts: mixing of liquid ingredients and mixing dry ingredients. The liquid ingredients (eggs and vanilla) were mixed together at high speed for 3 min using an electrical mixer (Kenwood 800 New Lane, Havant, UK), then oil and warm water at 40 °C (pre-mixed with yeast) were added to the mixture. The flour and other dry ingredients were mixed together and added slowly to the liquid mixture and mixed together slowly to develop soft dough. The dough was left to rest for 30 min at room temperature.

#### 2.3.2. Filling

The brown sugar, cut walnuts and ground cinnamon were mixed together for the filling of the dough. The dough was fermented at 35 °C for 30 min and the butter was spread to cover all the dough, then the previous mixture was put on the top of the butter and the dough. The filled dough was rolled and cut manually to make 25 small pieces.

#### 2.3.3. Baking

Finally, the cinnamon rolls were baked to a desirable texture using a baking oven (Rational model CD61; Landsberg am Lech; Germany) at 180 °C for 20 min. Then the baked cinnamon rolls were removed from the oven and allowed to cool at room temperature for 10–15 min. The glaze ingredients mixed together (sweet concentrated milk and ground cinnamon) were spread on the top of the baked cinnamon rolls.

### 2.4. Chemical Analysis

The Standard AOAC methods were used for determining moisture, ash, crude fiber, protein and fat contents [16]. The total carbohydrates were calculated as 100 − (protein + fat + moisture + ash + fiber). The chemical procedures were performed on both the flours and final products.

#### Unsaturated Fatty Acids Determination

Unsaturated fatty acids content of the finished product was conducted for treatments 2, 5, 7 and control sample. The measurement of the unsaturated fatty acids content was performed following the method of Trattner et al. (2015) [17].

### 2.5. Physical Analysis

The diameter, thickness and weight of the rolls were determined [18]. The diameter of the cinnamon rolls was measured using a right-angle ruler. The ruler tapped on the side of the cinnamon rolls and the measure was taken in centimeters. The thickness of the cinnamon rolls was obtained by using a ruler. The ruler was placed from the head to the bottom of the cinnamon rolls and the measurement was taken in centimeters. The weight of the cinnamon roll samples was determined by using an electrical scale (SBA 31; Babenhausen, Germany).

### 2.6. Color Measurement

Minolat colorimeter CR-300 (Ramsey, NJ, USA) was used and recorded the L*a*b* color system. The L*a*b* color system consists of a luminance or lightness component (L*), (a* values) component for greenness (−a) to redness (+a) and the (b*) component from blueness (−b) to yellowness (+b). The colorimeter was calibrated by a light trap and white tile, according to a procedure set forth by the Minolat colorimeter manual. The color was measured at two different locations. The color measurements were taken in duplicate and then the data were averaged.

### 2.7. Trained Organoleptic Evaluation

A sensory evaluation test was performed to evaluate the quality and acceptability of the control and cinnamon roll treatments. Sixty consumers from different cultures and ages were involved in the sensory evaluation test for this study. The product evaluation criteria were used in training the consumers. The sensory evaluation was done for the flavor, texture, hardness, color, aftertaste and overall impression of all treatments. The scale that was utilized was 1 = dislike extremely to 7 = like extremely [19].

### 2.8. Statistical Analysis

Statistical Package for Social Sciences (SPSS, version15.0, 2007; Chicago, IL, USA) was used in the statistical analysis. The differences between the treatments followed by mean separation using Duncan’s analysis was performed by using SAS. A *p*-value of less than or equal to 0.05 was considered to be statistically significant. All measurements were taken in duplicates.

## 3. Results and Discussion

The results of the proximate analysis for raw flours (wheat flour, lupine flour, resistant starch and flaxseed flour) are shown in Table 4. The lupine and flaxseed flours contained a higher amount of ash (2.62 and 3.86%, respectively), protein (32.07 and 17.91%, respectively), lipid (6.03 and 34.60%, respectively) and insoluble fiber (16.21 and 16.53%, respectively) than wheat flour and resistant starch which had higher levels of carbohydrate (including soluble fibers) at 76.06 and 94.08%, respectively. Wheat flour, lupine flour, resistant starch and flaxseed flour had the highest level of moisture, protein, carbohydrate and lipids, respectively. Usually, wheat flour contains > 70% carbohydrates [20] which is consistent with the results of the current study. Similarly, it has been reported that lupine flour contains high levels of protein (30–40%) and low carbohydrate content [21]. Furthermore, it was reported that flaxseed flour used to prepare gluten-free bread contained a higher content of protein (40.0%), ash (6.9%) and fiber (34.0%), but lower lipid (8.8%) and carbohydrates (3.9%) levels, compared to flaxseed flour used in the current study [22].

**Figure 1:**
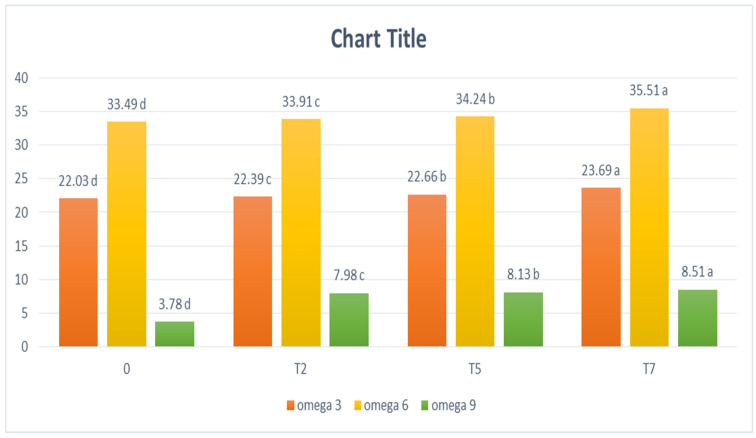
The unsaturated fatty acid content of the cinnamon rolls. Control 0: 100% wheat flour. Treatment 2: 80% resistant starch, 10% lupine flour, 10% flaxseed flour. Treatment 5: 65% resistant starch, 25% lupine flour, 10% flaxseed flour. Treatment 7: 55% resistant starch, 35% lupine flour, in addition to 10% flaxseed flour for all. Mean values with the same letters were not significantly different (*p* ≤ 0.05). Mean values with different letters were significantly different (*p* ≤ 0.05).

The proximate analysis results for the eight cinnamon roll treatments as well as control are shown in Table 5. Gluten-free cinnamon roll treatments were significantly higher in moisture, ash, lipid and insoluble fibers, due to higher contents of them in lupine and flaxseed flours. However, the protein content was lower in treatments 1, 2, 3 and 4 than control due to a low percentage of lupine flour. Moisture, ash, protein, lipid and insoluble fiber content of cinnamon roll treatments were increased significantly by increasing lupine flour. Moisture increase could be due to the presence of polar amino acids and the positive influence of increasing levels of protein on water-holding capacity as well as the increase in fiber content [23]. Accordingly, the reduction in the ability of wheat flour to bind water may be attributed to the low protein content [24]. Therefore, this could explain the reason that water absorption increased with an increase in protein content because lupine flour contains more protein than wheat flour [25]. The increase of ash, protein, lipid and soluble fiber is due to a high content of lupin flour. Moreover, the significant increase in protein content is attributed to the lupin and flaxseed flour, which contain about 40–45% protein [26] and about 28–30% protein [23], respectively. Figure 1 shows that treatment 2, 5 and 7 gluten-free cinnamon rolls had a higher content of omega 3, 6 and 9 than control because of its content of flaxseed which is considered the richest source of omega 3 fatty acids among non-seafood-related products and contains high amount of omega 6 fatty acids [27]. It was reported that flaxseed oil had the highest level of essential polyunsaturated fatty acids (71.8%) which are linolenic acid (55%) as omega 3 fatty acid and linoleic acid (16.8%) as omega 6 fatty acid [22]. Furthermore, these treatments also contain lupine flour that can be considered as a good source of omega 3, 6 and 9 fatty acids. Lupine flour contains 34.6% polyunsaturated fatty acids with 7.5% omega 3 fatty acids and 27.1% omega 6 fatty acids [23].

### 3.1. Physical Characteristics

Table 6 shows the physical characteristics of the different treatments in addition to the control. In general, all gluten-free cinnamon rolls had a significant increase in weight compared to the control sample. This result is similar to previous studies which used lupine flour to prepare gluten-free bakery products [28,29]. The increase in weight could be due to the high fiber and protein content of the gluten-free samples since fiber and protein are characterized by their high water-holding capacity, which leads to an increase in weight [21]. On the other hand, the cinnamon roll samples had slight differences in thickness and diameter values which may be due to manual cutting. However, the absence of gluten protein may lead to lower gas retention and lower dough expansion which could reduce the thickness and the diameter of the final product [30].

### 3.2. Color Measurements

**Figure 2:**
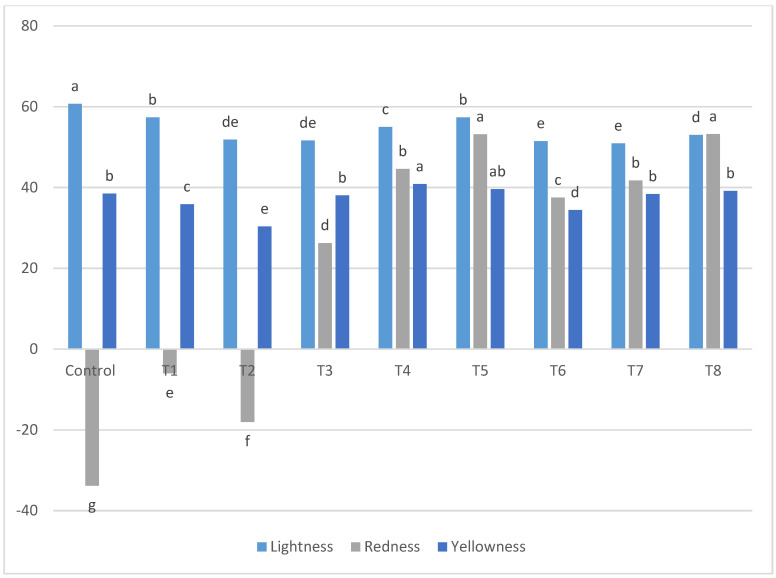
The mean of (L*) lightness, (a*) redness and (b*) yellowness values of gluten-free and C, control: 100% wheat flour; T1–T8 are presented by 85:5, 80:10, 75:15, 70:20, 65:25, 60:30, 55:35 and 50:40 as % of resistant starch to lupin in the presence of 10% flaxseed flour for all the treatments. Column values with the same letters were not significantly different (*p* ≤ 0.05). Means with different letters in the same column are significant at (*p* ≤ 0.05).

Color is the first factor in determining a bakery product’s quality by consumers [31]. Naturally, lupine flour is more yellow than wheat flour; therefore, substitution of wheat flour with lupin flour in a prepared formula strongly affected the cinnamon rolls’ color. As shown in Figure 2, there were significant (L*), (a*) and (b*) color values between the gluten-free samples and the control. These differences in the color characteristics may be due to the differences in the colors of the flour pigments which in turns depend on the plant origin [32]. However, the differences in color values among the cinnamon roll samples may be due to the variation in the percentage of the composite flour for each treatment. Treatments containing a higher level of lupine flour had higher redness values due to the higher protein level that causes the incidence of the Maillard reaction [33]. Moreover, treatments containing a higher amount of resistant starch had lower redness due to a lower content of protein [34,35]. In general, the lightness decreased as the lupine flour increased. However, inconsistent patterns were observed in the yellowness of cinnamon rolls which may be due to the effect of the ingredients used in the glaze. Similarly, it was found that the lightness value of muffins decreased as lupine flour increased [36]. A reduction in the lightness of cookies prepared with different cereals (rice, buckwheat, oat, kamut and spelt) was also found, and enriched with 10% lupin compared with control samples [37].

### 3.3. Sensory Evaluation

**Figure 3:**
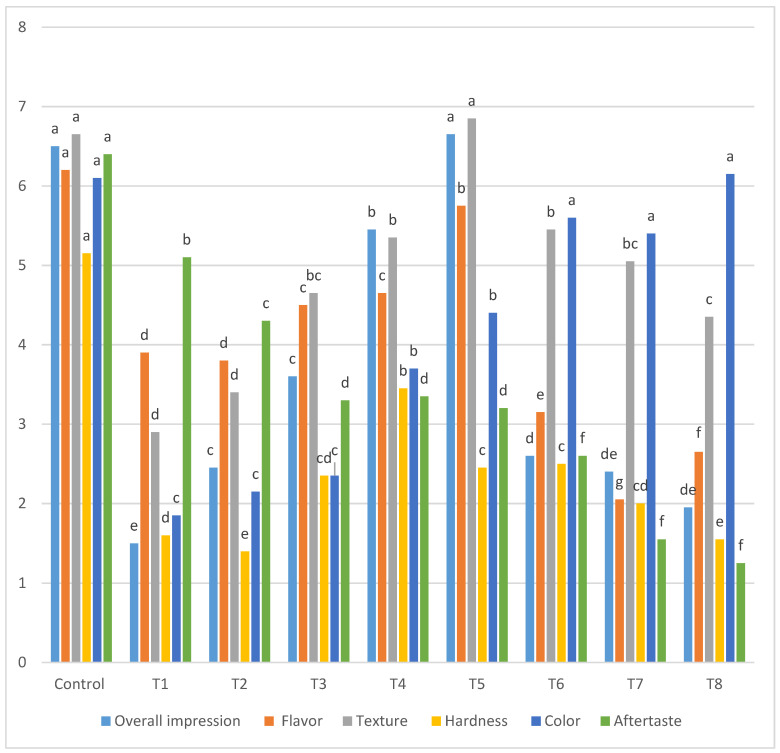
Sensory evaluation (overall impression, flavor, texture, hardness, color and aftertaste) for gluten-free cinnamon roll treatments and control (100% wheat flour); T1–T8 are presented by 85:5, 80:10, 75:15, 70:20, 65:25, 60:30, 55:35 and 50:40 as % of resistant starch to lupin in the presence of 10% flaxseed flour for all the treatments. Column values with the same letters were not significantly different (*p* ≤ 0.05). Means with different letters in the same column are significant at (*p* ≤ 0.05).

The acceptance of the sensory quality is a crucial step for the success of newly developed products. Therefore, an important factor in the novel product development is to measure its organoleptic characteristics as they play an essential role in the determining final consumer preference. In this study, the evaluators were asked to assess the sensory parameters of the control and gluten-free cinnamon roll treatments (T1–T8) including overall impression, flavor, texture, hardness, color and aftertaste. The sensory results of the cinnamon rolls are presented in Figure 3. The rate of overall impression, flavor and aftertaste decreased significantly with an increase in the lupine flour and decrease in resistant starch. The results are in agreement with various researchers [37,38], who suggested that the beany flavor in legumes flour could be the reason for the previously mentioned attributes receiving lower scores by the evaluators. With respect to the color attribute, the cinnamon roll color was improved by the addition of lupin flour from T6–T8. The combination effect of different concentrations of resistant starch with lupin at these levels may be the main reason behind this variation in the evaluation scores. However, a light yellow color given by the natural yellow color of lupin flour was appealing to the evaluators at the highest concentration (T8). This result was in agreement with the findings of other researchers [36,39]. With respect to the texture attributes, the texture was improved with incorporation of lupine flour and resistant starch up to 25% and 65%, respectively. This could indicate the advantage behind adding these two components to the treatments at these levels and their combined effect. Moreover, the highest hardness scores were obtained through the combined effect of lupin flour and resistant starch for T4 (20%:70%). The control sample significantly (*p* ≤ 0.05) received the highest scores in overall impression, flavor, hardness and aftertaste in comparison with all gluten-free treatments. Treatment 5 with 60% resistant starch, 30% of lupine flour and 10% flaxseed significantly (*p* ≤ 0.05) received the highest score in overall impression, flavor, texture and color among gluten-free cinnamon roll treatments.

## 4. Conclusions

The results of the study were promising such that it is feasible to develop a quality gluten-free cinnamon roll with respect to nutritional value manifested in higher levels of protein, fibers, unsaturated acids and prebiotics and acceptable sensory attributes by replacing wheat flour with resistant starch, lupine and flaxseed flours. The recipe that received the best quality and organoleptic characteristics was composed of 65% resistant starch and 25% lupine flour with 10% flaxseed flour and 1% xanthan gum.

## Figures and Tables

**Table 1:** Control cinnamon roll ingredients.

Ingredients	%
Wheat flour	44.8
Egg	9.5
Soy bean oil	6.8
Dry full fat milk	13.6
Sugar	6.8
Yeast	1.25
Baking soda	0.27
Vanilla	0.68
Water	16.3

**Table 2:** Filling ingredients of cinnamon rolls.

Ingredients	%
Brown sugar	42.5
Ground cinnamon	7.5
Butter	25
Walnut	25

**Table 3:** Glaze ingredients of cinnamon rolls.

Ingredients	%
Sweet concentrated milk	95.2
Ground cinnamon	4.8

**Table 4:** The percentage of moisture, ash, protein, lipid, carbohydrate (including soluble fibers) and insoluble fibers in wheat flour, resistant starch, lupine flour and flaxseed flour.

Flours	Moisture % *	Ash % *	Protein % *	Lipid % *	Carbohydrate (Including Soluble Fibers) % *	Fiber % *
Wheat flour	11.92 ± 0.01 ^a^	0.44 ± 0.06 ^c^	9.87 ± 0.24 ^c^	1.32 ± 0.23 ^c^	76.06 ± 2.09 ^b^	0.39 ± 0.17 ^d^
Resistant starch	5.12 ± 0.03 ^d^	0.18 ± 0.03 ^d^	0.06 ± 0.01 ^d^	0.15 ± 0.05 ^d^	94.08 ± 0.02 ^a^	0.41± 0.01 ^c^
Lupine flour	8.31 ± 0.08 ^b^	2.62 ± 0.03 ^b^	32.07 ± 0.09 ^a^	6.03 ± 0.04 ^b^	34.76 ± 0.08 ^c^	16.21 ± 0.01 ^b^
Flaxseed flour	6.40 ± 0.01 ^c^	3.86 ± 0.01 ^a^	17.91 ± 0.01 ^b^	34.60 ± 0.00 ^a^	20.70 ± 0.01 ^d^	16.53 ± 0.03 ^a^

Column values with the same letters were not significantly different at (*p* ≤ 0.05). Column values with different letters were significantly different at (*p* ≤ 0.05). * These values are the average of two replicates ± the standard deviation.

**Table 5:** The percentage of moisture, ash, protein, lipid, carbohydrate (including soluble fibers) and insoluble fibers of the control sample and the treatments.

Treatment	Moisture % *	Ash % *	Protein % *	Lipid % *	Carbohydrate (Including Soluble Fiber) % *	Fiber % *
C	15.87 ± 0.01 ^h^	1.17 ± 0.01 ^f^	8.22 ± 0.21 ^e^	18.04 ± 0.04 ^g^	54.74 ± 0.04 ^c^	1.96 ± 0.04 ^h^
T1	16.40 ± 0.08 ^g^	1.33 ± 0.08 ^e^	3.20 ± 0.02 ^i^	19.35 ± 0.01 ^f^	56.11 ± 0.06 ^a^	3.61 ± 0.03 ^g^
T2	16.59 ± 0.05 ^g^	1.35 ± 0.02 ^e^	4.03 ± 0.03 ^h^	19.37 ± 0.01 ^f^	54.95 ± 0.03 ^b^	3.71 ± 0.01 ^g^
T3	16.72 ± 0.03 ^f^	1.41 ± 0.01 ^de^	5.32 ± 0.01 ^g^	19.51 ± 0.03 ^e^	53.00 ± 0.04 ^d^	4.04 ± 0.03 ^f^
T4	16.83 ± 0.01 ^e^	1.49 ± 0.01 ^cd^	7.02 ± 0.05 ^f^	19.59 ± 0.03 ^d^	50.90 ± 0.06 ^e^	4.17 ± 0.02 ^e^
T5	17.00 ± 0.01 ^d^	1.54 ± 0.01 ^c^	9.03 ± 0.03 ^d^	19.65 ± 0.01 ^d^	48.08 ± 0.03 ^f^	4.70 ± 0.02 ^d^
T6	17.89 ± 0.05 ^c^	1.60 ± 0.04 ^b^	10.45 ± 0.06 ^c^	20.02 ± 0.01 ^c^	44.63 ± 0.03 ^g^	5.41 ± 0.02 ^c^
T7	18.57 ± 0.02 ^b^	1.64 ± 0.03 ^b^	11.74 ± 0.04 ^b^	20.72 ± 0.02 ^b^	41.31 ± 0.03 ^h^	6.02 ± 0.03 ^b^
T8	19.43 ± 0.02 ^a^	1.74 ± 0.04 ^a^	12.31 ± 0.02 ^a^	21.16 ± 0.01 ^a^	38.52 ± 0.04 ^i^	6.84 ± 0.06 ^a^

Column values with the same letters were not significantly different (*p* ≤ 0.05). Means with different letters in the same column are significant at (*p* ≤ 0.05). * Values are the average of two replicates ± the standard deviation. C, control: 100% wheat flour; T1–T8 are presented by 85:5, 80:10, 75:15, 70:20, 65:25, 60:30, 55:35 and 50:40 as % of resistant starch and lupin in the presence of 10% flaxseed flour for all the treatments.

**Table 6:** The mean value of weight, diameter and thickness of different gluten-free cinnamon roll treatments and control.

Treatment	Weight (g) *	Diameter (cm) *	Thickness (cm) *
C	26.25 ± 0.49 ^c^	5.55 ± 0.07 ^a^	1.65 ± 0.21 ^ab^
T1	27.80 ± 0.30 ^bc^	5.65 ± 0.07 ^a^	1.60 ± 0.00 ^ab^
T2	28.75 ± 0.35 ^abc^	5.65 ± 0.07 ^a^	1.60 ± 0.14 ^ab^
T3	29.35 ± 1.20 ^abc^	5.50 ± 0.00 ^a^	1.60 ± 0.3 ^ab^
T4	22.05 ± 0.35 ^d^	5.05 ± 0.07 ^b^	1.40 ± 0.00 ^b^
T5	30.75 ± 1.06 ^a^	5.10 ± 0.14 ^b^	1.80 ± 0.00 ^a^
T6	31.50 ± 3.53 ^a^	5.6 ± 0.14 ^a^	1.55 ± 0.07 ^ab^
T7	27.70 ± 0.28 ^bc^	5.05 ± 0.21 ^b^	1.50 ± 0.00 ^ab^
T8	27.83 ± 0.60 ^bc^	4.95 ± 0.07 ^b^	1.75 ± 0.07 ^a^

Column values with the same letters were not significantly different (*p* ≤ 0.05). Means with different letters in the same column are significant at (*p* ≤ 0.05). * Values are the average of two replicates ± the standard deviation. C, control: 100% wheat flour; T1–T8 are presented by 85:5, 80:10, 75:15, 70:20, 65:25, 60:30, 55:35 and 50:40 as % of resistant starch to lupin in the presence of 10% flaxseed flour for all the treatments.
